# *catena*-Poly[[[(2,2′-bipyridine-κ^2^ *N*,*N*′)(dimethyl sulfoxide-κ*O*)(nitrato-κ^2^ *O*,*O*′)bis­muth(III)]-μ-5-carb­oxy­benzene-1,3-dicarboxyl­ato-κ^4^ *O* ^1^ *,O* ^1′^ *:O* ^3^ *,O* ^3′^] dimethyl sulfoxide monosolvate]

**DOI:** 10.1107/S1600536811005745

**Published:** 2011-02-19

**Authors:** Hoda Pasdar, Marjan Namegh, Hossein Aghabozorg, Behrouz Notash

**Affiliations:** a Department of Chemistry, Islamic Azad University, North Tehran Branch, Tehran, Iran; b Department of Chemistry, Shahid Beheshti University, G. C., Evin, Tehran 1983963113, Iran

## Abstract

The polymeric title compound, {[Bi(C_9_H_4_O_6_)(NO_3_)(C_10_H_8_N_2_)(C_2_H_6_OS)]·C_2_H_6_OS}_*n*_, was obtained by the reaction of bis­muth(III) nitrate, bipyridine (bpy) and 1,3,5-benzene­tricarb­oxy­lic acid (H_3_BTC). The Bi^III^ ion is coordinated in a distorted tricapped trigonal-prismatic geometry, defined by two N atoms of the bipy ligand, four O atoms of two HBTC^2−^ anions, two O atoms of a nitrate anion and one O atom of a dimethyl sulfoxide ligand. The crystal packing is stabilized by O—H⋯O and C—H⋯O hydrogen bonds. The S atom of the non-coordinating dimethyl sulfoxide mol­ecule is disordered over two sets of sites with refined site-occupancies of 0.430 (19) and 0.570 (19).

## Related literature

For coordination polymers derived from H_3_BTC, see: Skakle *et al.* (2001 ▶); Cheng *et al.* (2009 ▶). For related structures, see: Barbour *et al.* (1998 ▶); Bowmaker *et al.* (1998 ▶).
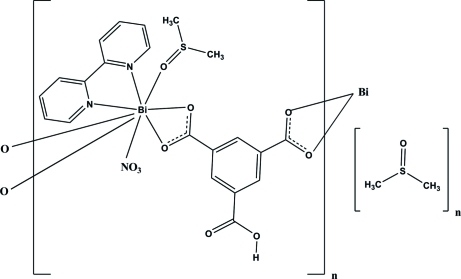


## Experimental

### 

#### Crystal data

[Bi(C_9_H_4_O_6_)(NO_3_)(C_10_H_8_N_2_)(C_2_H_6_OS)]·C_2_H_6_OS
*M* *_r_* = 791.57
Triclinic, 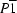

*a* = 8.9562 (18) Å
*b* = 9.882 (2) Å
*c* = 16.111 (3) Å
α = 89.78 (3)°
β = 76.22 (3)°
γ = 84.80 (3)°
*V* = 1378.9 (5) Å^3^
*Z* = 2
Mo *K*α radiation
μ = 6.61 mm^−1^
*T* = 298 K
0.15 × 0.10 × 0.1 mm

#### Data collection

STOE IPDS II diffractometer
Absorption correction: numerical [shape of crystal determined optically (*X-SHAPE* and *X-RED32*; Stoe & Cie (2005 ▶)] *T*_min_ = 0.455, *T*_max_ = 0.515
15461 measured reflections
7396 independent reflections
6043 reflections with *I* > 2σ(*I*)
*R*_int_ = 0.124

#### Refinement

*R*[*F* ^2^ > 2σ(*F* ^2^)] = 0.072
*wR*(*F* ^2^) = 0.195
*S* = 1.13
7396 reflections
373 parameters
H-atom parameters constrained
Δρ_max_ = 2.25 e Å^−3^
Δρ_min_ = −2.57 e Å^−3^

### 

Data collection: *X-AREA* (Stoe & Cie, 2005 ▶); cell refinement: *X-AREA*; data reduction: *X-AREA*; program(s) used to solve structure: *SHELXS97* (Sheldrick, 2008 ▶); program(s) used to refine structure: *SHELXL97* (Sheldrick, 2008 ▶); molecular graphics: *ORTEP-3 for Windows* (Farrugia, 1997 ▶); software used to prepare material for publication: *WinGX* (Farrugia, 1999 ▶).

## Supplementary Material

Crystal structure: contains datablocks I, global. DOI: 10.1107/S1600536811005745/bt5453sup1.cif

Structure factors: contains datablocks I. DOI: 10.1107/S1600536811005745/bt5453Isup2.hkl

Additional supplementary materials:  crystallographic information; 3D view; checkCIF report

## Figures and Tables

**Table 1:** Hydrogen-bond geometry (Å, °)

*D*—H⋯*A*	*D*—H	H⋯*A*	*D*⋯*A*	*D*—H⋯*A*
O1—H1⋯O11^i^	0.82	1.79	2.60 (2)	167
C10—H10⋯O8	0.93	2.53	3.16 (2)	125
C12—H12⋯O10^ii^	0.93	2.58	3.51 (2)	179
C13—H13⋯O6^iii^	0.93	2.57	3.350 (19)	142
C17—H17⋯O2^iii^	0.93	2.48	3.260 (19)	142
C18—H18⋯O3^iv^	0.93	2.58	3.391 (17)	146
C21—H21*B*⋯O9^v^	0.96	2.56	3.49 (3)	161

Symmetry codes: (i) 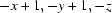; (ii) 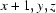; (iii) 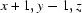; (iv) 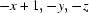; (v) 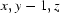.

**Fig. 1.**
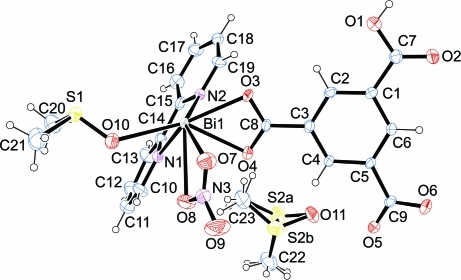
The asymmetric unit of title compound with displacement ellipsoids drawn at 30% probability level.

**Fig. 2.**
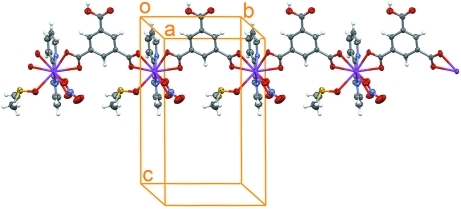
A view of the one-dimensional coordination polymer of the title compound. Uncoordinated DMSO molecules have been omitted for clarity.

## supplementary crystallographic information

### Comment

Coordination polymers are currently of great interest due to structural versatility, unique properties and their applications in different area of science. 1,3,5-benzenetricarboxylic acid (H_3_BTC) has three carboxylate groups and it can form coordination polymers with wide range of dimensionality from one-dimensional to three-dimensional.

The asymmetric unit of the title compound is presented in Fig. 1. In the the title compound, Bi^III^ ion is 9-coordinated in a N_2_O_7_ environment and its geometry is distorted tricapped trigonal prismatic. In the title compound, there is one H_3_BTC in which two carboxylic groups are deprotonate. There are also one bipyridine, one nitrate and one dimethylsulfoxide molecule. Also there is one uncoordinated DMSO molecule in the packing of the compound. Bond lengths are within normal ranges. The crystal structure of title compound shows that the compound is extended by HBTC^2-^ moieties and it is a one-dimensional coordination polymer. The polymeric structure of title compound is presented in Fig. 2. There are several O—H···O and C—H···O hydrogen bonds in the structure of the title compound which stabilize crystal packing (Table 1).

### Experimental

A solution of Bi(NO_3_)_3_.5H_2_O (0.5 mmol, 0.2425 g) in DMSO (5 ml) was added to a mixture of bipyridine (1 mmol, 0.1569 g) and 1,3,5-benzenetricarboxylic acid (1 mmol,0.2101 g) in absolute ethanol (10 ml) and stirred for 2 hrs at room temperature. After 5 months, colorless crystals of the title compound appeared (m.p: 270°C decompose).

### Refinement

All hydrogen atoms were positioned geometrically and refined as riding atoms with C—H = 0.93 Å and *U*iso(H) = 1.2 *U*eq(C) for aromatic C—H groups, C—H = 0.96 Å and *U*iso(H) = 1.5 *Ueq*(C) for methyl groups and O—H = 0.82 Å with *U*iso(H) = 1.5 *Ueq*(O). The sulfur atom of uncoordinated DMSO is disordered over two positions with refined site-occupancies of 0.430 (19) and 0.570 (19).

### Crystal data

**Table:**

[Bi(C_9_H_4_O_6_)(NO_3_)(C_10_H_8_N_2_)(C_2_H_6_OS)]·C_2_H_6_OS	*Z* = 2
*M**_r_* = 791.57	*F*(000) = 772.0
Triclinic, *P*1	*D*_x_ = 1.906 Mg m^−^^3^
Hall symbol: -P 1	Mo *K*α radiation, λ = 0.71073 Å
*a* = 8.9562 (18) Å	Cell parameters from 7396 reflections
*b* = 9.882 (2) Å	θ = 2.4–29.3°
*c* = 16.111 (3) Å	µ = 6.61 mm^−^^1^
α = 89.78 (3)°	*T* = 298 K
β = 76.22 (3)°	Plate, colorless
γ = 84.80 (3)°	0.15 × 0.1 × 0.1 mm
*V* = 1378.9 (5) Å^3^	

### Data collection

**Table:**

STOE IPDS II diffractometer	7396 independent reflections
Radiation source: fine-focus sealed tube	6043 reflections with *I* > 2σ(*I*)
graphite	*R*_int_ = 0.124
Detector resolution: 0.15 mm pixels mm^-1^	θ_max_ = 29.3°, θ_min_ = 2.4°
rotation method scans	*h* = −12→12
Absorption correction: numerical [shape of crystal determined optically (*X-SHAPE* and *X-RED32*; Stoe & Cie (2005)]	*k* = −13→13
*T*_min_ = 0.455, *T*_max_ = 0.515	*l* = −22→22
15461 measured reflections	

### Refinement

**Table:**

Refinement on *F*^2^	Primary atom site location: structure-invariant direct methods
Least-squares matrix: full	Secondary atom site location: difference Fourier map
*R*[*F*^2^ > 2σ(*F*^2^)] = 0.072	Hydrogen site location: inferred from neighbouring sites
*wR*(*F*^2^) = 0.195	H-atom parameters constrained
*S* = 1.13	*w* = 1/[σ^2^(*F*_o_^2^) + (0.0865*P*)^2^ + 12.3704*P*] where *P* = (*F*_o_^2^ + 2*F*_c_^2^)/3
7396 reflections	(Δ/σ)_max_ < 0.001
373 parameters	Δρ_max_ = 2.25 e Å^−^^3^
0 restraints	Δρ_min_ = −2.57 e Å^−^^3^

### Special details

**Table:**

Geometry. All e.s.d.'s (except the e.s.d. in the dihedral angle between two l.s. planes) are estimated using the full covariance matrix. The cell e.s.d.'s are taken into account individually in the estimation of e.s.d.'s in distances, angles and torsion angles; correlations between e.s.d.'s in cell parameters are only used when they are defined by crystal symmetry. An approximate (isotropic) treatment of cell e.s.d.'s is used for estimating e.s.d.'s involving l.s. planes.
Refinement. Refinement of *F*^2^ against ALL reflections. The weighted *R*-factor *wR* and goodness of fit *S* are based on *F*^2^, conventional *R*-factors *R* are based on *F*, with *F* set to zero for negative *F*^2^. The threshold expression of *F*^2^ > σ(*F*^2^) is used only for calculating *R*-factors(gt) *etc*. and is not relevant to the choice of reflections for refinement. *R*-factors based on *F*^2^ are statistically about twice as large as those based on *F*, and *R*- factors based on ALL data will be even larger.

### Fractional atomic coordinates and isotropic or equivalent isotropic displacement parameters (Å^2^)

**Table:**

	*x*	*y*	*z*	*U*_iso_*/*U*_eq_	Occ. (<1)
S2B	0.8840 (10)	0.4785 (8)	0.2723 (6)	0.061 (3)	0.570 (19)
S2A	0.9839 (16)	0.4592 (14)	0.2253 (8)	0.072 (4)	0.430 (19)
Bi1	0.45530 (5)	0.03366 (4)	0.26887 (3)	0.02732 (13)	
S1	0.3345 (4)	−0.2775 (3)	0.3990 (2)	0.0452 (7)	
O1	0.2022 (16)	0.4330 (11)	−0.0595 (8)	0.065 (3)	
H1	0.1625	0.4435	−0.1003	0.097*	
O2	0.1908 (14)	0.6580 (10)	−0.0621 (6)	0.054 (3)	
O3	0.3771 (11)	0.1788 (7)	0.1652 (6)	0.0390 (19)	
O4	0.5178 (12)	0.2701 (8)	0.2386 (6)	0.0407 (19)	
O5	0.5247 (11)	0.7833 (8)	0.2306 (5)	0.0374 (18)	
O6	0.3481 (9)	0.8965 (7)	0.1763 (6)	0.0351 (17)	
O7	0.2078 (14)	0.1922 (12)	0.3655 (8)	0.061 (3)	
O8	0.4030 (14)	0.1866 (12)	0.4194 (7)	0.059 (3)	
O9	0.215 (2)	0.3348 (16)	0.4667 (10)	0.100 (6)	
O10	0.3402 (14)	−0.1262 (10)	0.4050 (6)	0.053 (2)	
O11	0.898 (3)	0.5681 (15)	0.1982 (11)	0.116 (7)	
N1	0.7024 (12)	−0.0147 (9)	0.3099 (6)	0.0336 (19)	
N2	0.6774 (11)	0.0000 (9)	0.1469 (6)	0.0308 (18)	
N3	0.2718 (18)	0.2378 (12)	0.4173 (8)	0.054 (3)	
C1	0.2933 (12)	0.5408 (10)	0.0437 (6)	0.0268 (19)	
C2	0.3340 (13)	0.4186 (10)	0.0780 (7)	0.030 (2)	
H2	0.3138	0.3382	0.0548	0.036*	
C3	0.4043 (13)	0.4129 (10)	0.1462 (7)	0.030 (2)	
C4	0.4407 (14)	0.5316 (10)	0.1788 (7)	0.032 (2)	
H4	0.4945	0.5279	0.2217	0.039*	
C5	0.3958 (14)	0.6593 (10)	0.1468 (6)	0.030 (2)	
C6	0.3241 (14)	0.6622 (10)	0.0792 (7)	0.032 (2)	
H6	0.2963	0.7452	0.0570	0.038*	
C7	0.2216 (14)	0.5520 (12)	−0.0301 (8)	0.036 (2)	
C8	0.4372 (15)	0.2817 (10)	0.1852 (7)	0.033 (2)	
C9	0.4271 (13)	0.7856 (10)	0.1874 (7)	0.030 (2)	
C10	0.7121 (18)	−0.0062 (15)	0.3920 (8)	0.047 (3)	
H10	0.6233	0.0173	0.4346	0.057*	
C11	0.852 (2)	−0.0318 (16)	0.4135 (11)	0.056 (4)	
H11	0.8568	−0.0244	0.4703	0.068*	
C12	0.982 (2)	−0.0681 (18)	0.3517 (12)	0.062 (4)	
H12	1.0779	−0.0824	0.3651	0.074*	
C13	0.9688 (18)	−0.0835 (17)	0.2665 (10)	0.054 (4)	
H13	1.0547	−0.1136	0.2236	0.065*	
C14	0.8294 (14)	−0.0539 (12)	0.2481 (8)	0.037 (2)	
C15	0.8066 (15)	−0.0620 (11)	0.1590 (8)	0.038 (2)	
C16	0.9191 (15)	−0.1297 (15)	0.0941 (9)	0.045 (3)	
H16	1.0095	−0.1729	0.1043	0.054*	
C17	0.8889 (18)	−0.1293 (15)	0.0133 (9)	0.048 (3)	
H17	0.9598	−0.1751	−0.0317	0.058*	
C18	0.7567 (19)	−0.0627 (13)	−0.0014 (8)	0.046 (3)	
H18	0.7385	−0.0598	−0.0559	0.055*	
C19	0.6523 (14)	−0.0003 (13)	0.0673 (8)	0.037 (2)	
H19	0.5608	0.0433	0.0588	0.045*	
C20	0.512 (2)	−0.359 (2)	0.4063 (13)	0.071 (5)	
H20A	0.5929	−0.3282	0.3615	0.107*	
H20B	0.5108	−0.4554	0.4005	0.107*	
H20C	0.5315	−0.3378	0.4607	0.107*	
C21	0.223 (3)	−0.319 (2)	0.5040 (13)	0.091 (7)	
H21A	0.2691	−0.2860	0.5470	0.137*	
H21B	0.2219	−0.4163	0.5081	0.137*	
H21C	0.1192	−0.2781	0.5123	0.137*	
C22	1.032 (3)	0.514 (2)	0.3181 (14)	0.078 (6)	
H22A	1.0919	0.4415	0.3387	0.118*	0.57
H22B	0.9395	0.5394	0.3611	0.118*	0.57
H22C	1.0915	0.5907	0.3053	0.118*	0.57
H22D	0.9901	0.6062	0.3314	0.118*	0.43
H22E	1.1425	0.5084	0.3090	0.118*	0.43
H22F	0.9905	0.4570	0.3647	0.118*	0.43
C23	0.906 (6)	0.315 (3)	0.251 (2)	0.17 (2)	
H23A	0.8952	0.2659	0.3031	0.254*	0.57
H23B	1.0072	0.2916	0.2148	0.254*	0.57
H23C	0.8294	0.2923	0.2220	0.254*	0.57
H23D	0.9260	0.3006	0.1901	0.254*	0.43
H23E	0.8140	0.2750	0.2785	0.254*	0.43
H23F	0.9918	0.2742	0.2712	0.254*	0.43

### Atomic displacement parameters (Å^2^)

**Table:**

	*U*^11^	*U*^22^	*U*^33^	*U*^12^	*U*^13^	*U*^23^
S2B	0.053 (5)	0.069 (5)	0.060 (5)	0.007 (3)	−0.018 (4)	−0.008 (3)
S2A	0.066 (8)	0.085 (8)	0.061 (7)	−0.009 (6)	−0.009 (6)	0.021 (6)
Bi1	0.0362 (2)	0.01789 (17)	0.0299 (2)	−0.00129 (12)	−0.01221 (14)	0.00062 (12)
S1	0.062 (2)	0.0341 (14)	0.0411 (16)	−0.0055 (13)	−0.0141 (15)	0.0042 (12)
O1	0.107 (10)	0.044 (5)	0.059 (6)	−0.011 (6)	−0.050 (7)	0.007 (5)
O2	0.085 (8)	0.037 (5)	0.045 (5)	0.012 (5)	−0.031 (5)	−0.001 (4)
O3	0.064 (6)	0.018 (3)	0.044 (5)	−0.012 (3)	−0.027 (4)	0.005 (3)
O4	0.063 (6)	0.023 (3)	0.041 (4)	−0.002 (3)	−0.022 (4)	0.005 (3)
O5	0.052 (5)	0.026 (4)	0.037 (4)	−0.008 (3)	−0.015 (4)	0.001 (3)
O6	0.034 (4)	0.022 (3)	0.047 (5)	−0.004 (3)	−0.005 (3)	0.003 (3)
O7	0.055 (6)	0.063 (7)	0.070 (7)	0.005 (5)	−0.029 (6)	−0.008 (6)
O8	0.069 (7)	0.062 (6)	0.049 (6)	0.021 (5)	−0.029 (5)	−0.017 (5)
O9	0.126 (13)	0.084 (10)	0.087 (10)	0.046 (9)	−0.038 (9)	−0.053 (8)
O10	0.076 (7)	0.037 (5)	0.041 (5)	−0.005 (5)	−0.007 (5)	0.002 (4)
O11	0.23 (2)	0.062 (8)	0.100 (11)	−0.031 (11)	−0.122 (14)	0.028 (8)
N1	0.039 (5)	0.028 (4)	0.038 (5)	−0.006 (4)	−0.016 (4)	0.006 (4)
N2	0.041 (5)	0.022 (4)	0.031 (4)	0.000 (3)	−0.012 (4)	0.001 (3)
N3	0.078 (9)	0.040 (6)	0.042 (6)	0.004 (6)	−0.014 (6)	−0.003 (5)
C1	0.025 (5)	0.026 (4)	0.027 (5)	−0.006 (3)	−0.001 (4)	−0.003 (4)
C2	0.038 (6)	0.025 (4)	0.025 (5)	−0.004 (4)	−0.003 (4)	−0.006 (4)
C3	0.035 (5)	0.024 (4)	0.029 (5)	−0.003 (4)	−0.003 (4)	0.004 (4)
C4	0.049 (6)	0.020 (4)	0.026 (5)	0.003 (4)	−0.010 (4)	−0.001 (4)
C5	0.044 (6)	0.019 (4)	0.023 (4)	0.004 (4)	−0.003 (4)	−0.003 (3)
C6	0.040 (6)	0.023 (4)	0.035 (5)	0.004 (4)	−0.016 (5)	0.001 (4)
C7	0.038 (6)	0.034 (5)	0.034 (5)	−0.006 (4)	−0.007 (5)	0.004 (4)
C8	0.051 (7)	0.018 (4)	0.027 (5)	0.004 (4)	−0.006 (5)	0.002 (4)
C9	0.038 (6)	0.024 (4)	0.028 (5)	−0.011 (4)	−0.005 (4)	0.001 (4)
C10	0.056 (8)	0.060 (8)	0.027 (6)	0.001 (6)	−0.014 (5)	0.000 (5)
C11	0.076 (11)	0.051 (8)	0.051 (8)	−0.003 (7)	−0.034 (8)	0.000 (6)
C12	0.058 (9)	0.069 (10)	0.066 (10)	0.012 (8)	−0.035 (8)	0.009 (8)
C13	0.043 (7)	0.069 (10)	0.054 (8)	0.000 (7)	−0.019 (6)	0.004 (7)
C14	0.038 (6)	0.034 (5)	0.041 (6)	0.002 (4)	−0.014 (5)	0.004 (5)
C15	0.046 (7)	0.021 (4)	0.047 (7)	−0.002 (4)	−0.013 (5)	0.005 (4)
C16	0.033 (6)	0.053 (7)	0.042 (7)	−0.006 (5)	0.004 (5)	0.001 (6)
C17	0.051 (8)	0.048 (7)	0.043 (7)	0.004 (6)	−0.008 (6)	−0.012 (6)
C18	0.074 (10)	0.034 (6)	0.032 (6)	−0.016 (6)	−0.011 (6)	0.005 (5)
C19	0.036 (6)	0.046 (6)	0.035 (6)	−0.010 (5)	−0.016 (5)	−0.002 (5)
C20	0.086 (13)	0.067 (11)	0.071 (11)	0.004 (9)	−0.042 (10)	0.010 (9)
C21	0.14 (2)	0.066 (12)	0.061 (11)	−0.025 (13)	0.003 (12)	0.016 (9)
C22	0.080 (13)	0.095 (14)	0.078 (13)	−0.028 (11)	−0.047 (11)	0.026 (11)
C23	0.35 (6)	0.085 (18)	0.14 (3)	−0.06 (3)	−0.17 (4)	0.039 (18)

### Geometric parameters (Å, °)

**Table:**

S2B—O11	1.471 (16)	C4—H4	0.9300
S2B—C23	1.64 (3)	C5—C6	1.390 (15)
S2A—O11	1.395 (19)	C5—C9	1.490 (14)
S2A—C22	1.75 (2)	C6—H6	0.9300
Bi1—O3	2.386 (8)	C9—Bi1^ii^	2.844 (10)
Bi1—O6^i^	2.432 (9)	C10—C11	1.38 (2)
Bi1—N2	2.440 (10)	C10—H10	0.9300
Bi1—N1	2.463 (10)	C11—C12	1.37 (3)
Bi1—O4	2.472 (8)	C11—H11	0.9300
Bi1—O5^i^	2.537 (8)	C12—C13	1.42 (2)
Bi1—O7	2.745 (12)	C12—H12	0.9300
Bi1—O10	2.750 (10)	C13—C14	1.357 (18)
Bi1—C9^i^	2.844 (10)	C13—H13	0.9300
S1—O10	1.505 (10)	C14—C15	1.501 (18)
S1—C20	1.749 (19)	C15—C16	1.391 (18)
S1—C21	1.82 (2)	C16—C17	1.39 (2)
O1—C7	1.311 (16)	C16—H16	0.9300
O1—H1	0.8200	C17—C18	1.37 (2)
O2—C7	1.204 (14)	C17—H17	0.9300
O3—C8	1.268 (14)	C18—C19	1.374 (19)
O4—C8	1.248 (15)	C18—H18	0.9300
O5—C9	1.239 (14)	C19—H19	0.9300
O5—Bi1^ii^	2.537 (8)	C20—H20A	0.9600
O6—C9	1.286 (13)	C20—H20B	0.9600
O6—Bi1^ii^	2.432 (9)	C20—H20C	0.9600
O7—N3	1.226 (17)	C21—H21A	0.9600
O8—N3	1.245 (17)	C21—H21B	0.9600
O9—N3	1.241 (17)	C21—H21C	0.9600
N1—C14	1.348 (16)	C22—H22A	0.9600
N1—C10	1.349 (15)	C22—H22B	0.9600
N2—C15	1.316 (15)	C22—H22C	0.9600
N2—C19	1.354 (14)	C22—H22D	0.9600
C1—C2	1.380 (14)	C22—H22E	0.9600
C1—C6	1.407 (14)	C22—H22F	0.9600
C1—C7	1.479 (16)	C23—H23A	0.9600
C2—C3	1.390 (15)	C23—H23B	0.9600
C2—H2	0.9300	C23—H23C	0.9600
C3—C4	1.384 (15)	C23—H23D	0.9600
C3—C8	1.475 (13)	C23—H23E	0.9600
C4—C5	1.422 (13)	C23—H23F	0.9600
			
O11—S2B—C23	115.9 (14)	O6—C9—Bi1^ii^	58.4 (5)
O11—S2A—C22	106.9 (13)	C5—C9—Bi1^ii^	174.4 (8)
O3—Bi1—O6^i^	71.8 (3)	N1—C10—C11	120.9 (14)
O3—Bi1—N2	77.9 (3)	N1—C10—H10	119.5
O6^i^—Bi1—N2	80.2 (3)	C11—C10—H10	119.5
O3—Bi1—N1	133.0 (3)	C12—C11—C10	120.1 (14)
O6^i^—Bi1—N1	127.3 (3)	C12—C11—H11	120.0
N2—Bi1—N1	66.6 (3)	C10—C11—H11	120.0
O3—Bi1—O4	53.1 (3)	C11—C12—C13	118.3 (14)
O6^i^—Bi1—O4	123.8 (3)	C11—C12—H12	120.9
N2—Bi1—O4	78.6 (3)	C13—C12—H12	120.9
N1—Bi1—O4	89.2 (3)	C14—C13—C12	119.4 (15)
O3—Bi1—O5^i^	118.1 (3)	C14—C13—H13	120.3
O6^i^—Bi1—O5^i^	52.6 (3)	C12—C13—H13	120.3
N2—Bi1—O5^i^	68.7 (3)	N1—C14—C13	121.3 (12)
N1—Bi1—O5^i^	77.1 (3)	N1—C14—C15	116.1 (10)
O4—Bi1—O5^i^	147.4 (3)	C13—C14—C15	122.7 (12)
O3—Bi1—O7	77.4 (3)	N2—C15—C16	123.3 (12)
O6^i^—Bi1—O7	105.3 (3)	N2—C15—C14	116.0 (11)
N2—Bi1—O7	151.4 (3)	C16—C15—C14	120.6 (12)
N1—Bi1—O7	123.6 (3)	C15—C16—C17	116.4 (13)
O4—Bi1—O7	75.1 (4)	C15—C16—H16	121.8
O5^i^—Bi1—O7	137.0 (3)	C17—C16—H16	121.8
O3—Bi1—O10	142.1 (3)	C18—C17—C16	121.4 (13)
O6^i^—Bi1—O10	90.1 (3)	C18—C17—H17	119.3
N2—Bi1—O10	132.8 (3)	C16—C17—H17	119.3
N1—Bi1—O10	84.6 (3)	C17—C18—C19	117.5 (12)
O4—Bi1—O10	139.9 (3)	C17—C18—H18	121.2
O5^i^—Bi1—O10	68.9 (3)	C19—C18—H18	121.2
O7—Bi1—O10	75.7 (3)	N2—C19—C18	122.5 (12)
O3—Bi1—C9^i^	95.9 (3)	N2—C19—H19	118.7
O6^i^—Bi1—C9^i^	26.8 (3)	C18—C19—H19	118.7
N2—Bi1—C9^i^	73.5 (3)	S1—C20—H20A	109.5
N1—Bi1—C9^i^	102.1 (3)	S1—C20—H20B	109.5
O4—Bi1—C9^i^	142.3 (3)	H20A—C20—H20B	109.5
O5^i^—Bi1—C9^i^	25.8 (3)	S1—C20—H20C	109.5
O7—Bi1—C9^i^	123.3 (3)	H20A—C20—H20C	109.5
O10—Bi1—C9^i^	77.5 (3)	H20B—C20—H20C	109.5
O10—S1—C20	109.1 (8)	S1—C21—H21A	109.5
O10—S1—C21	103.5 (8)	S1—C21—H21B	109.5
C20—S1—C21	97.9 (11)	H21A—C21—H21B	109.5
C7—O1—H1	109.5	S1—C21—H21C	109.5
C8—O3—Bi1	95.3 (7)	H21A—C21—H21C	109.5
C8—O4—Bi1	91.8 (7)	H21B—C21—H21C	109.5
C9—O5—Bi1^ii^	91.1 (6)	S2A—C22—H22A	109.5
C9—O6—Bi1^ii^	94.8 (7)	S2A—C22—H22B	109.5
N3—O7—Bi1	98.6 (9)	H22A—C22—H22B	109.5
S1—O10—Bi1	124.3 (5)	S2A—C22—H22C	109.5
S2A—O11—S2B	42.0 (6)	H22A—C22—H22C	109.5
C14—N1—C10	120.0 (11)	H22B—C22—H22C	109.5
C14—N1—Bi1	118.2 (8)	S2A—C22—H22D	109.5
C10—N1—Bi1	121.7 (9)	H22A—C22—H22D	141.1
C15—N2—C19	118.8 (11)	H22B—C22—H22D	56.3
C15—N2—Bi1	118.5 (8)	H22C—C22—H22D	56.3
C19—N2—Bi1	118.6 (8)	S2A—C22—H22E	109.5
O7—N3—O9	123.7 (16)	H22A—C22—H22E	56.3
O7—N3—O8	118.7 (12)	H22B—C22—H22E	141.1
O9—N3—O8	117.6 (15)	H22C—C22—H22E	56.3
C2—C1—C6	118.9 (10)	H22D—C22—H22E	109.5
C2—C1—C7	123.6 (9)	S2A—C22—H22F	109.5
C6—C1—C7	117.5 (9)	H22A—C22—H22F	56.3
C1—C2—C3	121.6 (9)	H22B—C22—H22F	56.3
C1—C2—H2	119.2	H22C—C22—H22F	141.1
C3—C2—H2	119.2	H22D—C22—H22F	109.5
C4—C3—C2	119.6 (9)	H22E—C22—H22F	109.5
C4—C3—C8	119.8 (10)	S2B—C23—H23A	109.5
C2—C3—C8	120.5 (10)	S2B—C23—H23B	109.5
C3—C4—C5	119.9 (10)	H23A—C23—H23B	109.5
C3—C4—H4	120.0	S2B—C23—H23C	109.5
C5—C4—H4	120.0	H23A—C23—H23C	109.5
C6—C5—C4	119.1 (9)	H23B—C23—H23C	109.5
C6—C5—C9	122.3 (9)	S2B—C23—H23D	109.5
C4—C5—C9	118.6 (10)	H23A—C23—H23D	141.1
C5—C6—C1	120.7 (9)	H23B—C23—H23D	56.3
C5—C6—H6	119.7	H23C—C23—H23D	56.3
C1—C6—H6	119.7	S2B—C23—H23E	109.5
O2—C7—O1	123.5 (12)	H23A—C23—H23E	56.3
O2—C7—C1	123.9 (11)	H23B—C23—H23E	141.1
O1—C7—C1	112.4 (10)	H23C—C23—H23E	56.3
O4—C8—O3	119.5 (9)	H23D—C23—H23E	109.5
O4—C8—C3	122.1 (10)	S2B—C23—H23F	109.5
O3—C8—C3	118.4 (10)	H23A—C23—H23F	56.3
O5—C9—O6	121.4 (10)	H23B—C23—H23F	56.3
O5—C9—C5	121.2 (10)	H23C—C23—H23F	141.1
O6—C9—C5	117.4 (10)	H23D—C23—H23F	109.5
O5—C9—Bi1^ii^	63.1 (6)	H23E—C23—H23F	109.5
			
O6^i^—Bi1—O3—C8	−171.6 (8)	O5^i^—Bi1—N2—C19	90.6 (8)
N2—Bi1—O3—C8	−88.1 (8)	O7—Bi1—N2—C19	−67.0 (11)
N1—Bi1—O3—C8	−47.1 (9)	O10—Bi1—N2—C19	118.1 (8)
O4—Bi1—O3—C8	−3.3 (7)	C9^i^—Bi1—N2—C19	63.8 (8)
O5^i^—Bi1—O3—C8	−145.8 (7)	Bi1—O7—N3—O9	166.9 (16)
O7—Bi1—O3—C8	77.4 (8)	Bi1—O7—N3—O8	−11.3 (15)
O10—Bi1—O3—C8	123.1 (7)	C6—C1—C2—C3	0.0 (16)
C9^i^—Bi1—O3—C8	−159.7 (7)	C7—C1—C2—C3	178.5 (10)
O3—Bi1—O4—C8	3.4 (7)	C1—C2—C3—C4	−2.7 (17)
O6^i^—Bi1—O4—C8	16.8 (8)	C1—C2—C3—C8	175.9 (10)
N2—Bi1—O4—C8	86.7 (7)	C2—C3—C4—C5	4.7 (17)
N1—Bi1—O4—C8	152.9 (7)	C8—C3—C4—C5	−174.0 (10)
O5^i^—Bi1—O4—C8	88.7 (9)	C3—C4—C5—C6	−4.0 (17)
O7—Bi1—O4—C8	−81.9 (7)	C3—C4—C5—C9	175.8 (10)
O10—Bi1—O4—C8	−126.4 (7)	C4—C5—C6—C1	1.3 (17)
C9^i^—Bi1—O4—C8	43.9 (10)	C9—C5—C6—C1	−178.5 (10)
O3—Bi1—O7—N3	−123.6 (9)	C2—C1—C6—C5	0.7 (17)
O6^i^—Bi1—O7—N3	169.5 (9)	C7—C1—C6—C5	−178.0 (10)
N2—Bi1—O7—N3	−92.8 (11)	C2—C1—C7—O2	−177.3 (12)
N1—Bi1—O7—N3	10.0 (11)	C6—C1—C7—O2	1.2 (18)
O4—Bi1—O7—N3	−68.8 (9)	C2—C1—C7—O1	−0.5 (17)
O5^i^—Bi1—O7—N3	118.6 (9)	C6—C1—C7—O1	178.1 (11)
O10—Bi1—O7—N3	83.4 (9)	Bi1—O4—C8—O3	−5.8 (12)
C9^i^—Bi1—O7—N3	147.6 (8)	Bi1—O4—C8—C3	171.9 (10)
C20—S1—O10—Bi1	84.5 (10)	Bi1—O3—C8—O4	6.0 (12)
C21—S1—O10—Bi1	−172.0 (11)	Bi1—O3—C8—C3	−171.8 (9)
O3—Bi1—O10—S1	96.9 (8)	C4—C3—C8—O4	−11.0 (18)
O6^i^—Bi1—O10—S1	37.1 (8)	C2—C3—C8—O4	170.4 (11)
N2—Bi1—O10—S1	−39.5 (10)	C4—C3—C8—O3	166.8 (11)
N1—Bi1—O10—S1	−90.4 (8)	C2—C3—C8—O3	−11.8 (17)
O4—Bi1—O10—S1	−172.7 (6)	Bi1^ii^—O5—C9—O6	3.6 (11)
O5^i^—Bi1—O10—S1	−12.1 (7)	Bi1^ii^—O5—C9—C5	−176.0 (9)
O7—Bi1—O10—S1	142.9 (8)	Bi1^ii^—O6—C9—O5	−3.8 (11)
C9^i^—Bi1—O10—S1	13.4 (7)	Bi1^ii^—O6—C9—C5	175.8 (8)
C22—S2A—O11—S2B	−63.8 (12)	C6—C5—C9—O5	−160.4 (11)
C23—S2B—O11—S2A	−53 (2)	C4—C5—C9—O5	19.7 (16)
O3—Bi1—N1—C14	−53.6 (9)	C6—C5—C9—O6	20.0 (16)
O6^i^—Bi1—N1—C14	46.4 (9)	C4—C5—C9—O6	−159.9 (10)
N2—Bi1—N1—C14	−9.3 (8)	C14—N1—C10—C11	3(2)
O4—Bi1—N1—C14	−87.2 (8)	Bi1—N1—C10—C11	−179.1 (11)
O5^i^—Bi1—N1—C14	62.9 (8)	N1—C10—C11—C12	−1(2)
O7—Bi1—N1—C14	−158.7 (8)	C10—C11—C12—C13	−2(3)
O10—Bi1—N1—C14	132.4 (8)	C11—C12—C13—C14	4(3)
C9^i^—Bi1—N1—C14	56.5 (8)	C10—N1—C14—C13	−1.0 (19)
O3—Bi1—N1—C10	128.1 (10)	Bi1—N1—C14—C13	−179.3 (11)
O6^i^—Bi1—N1—C10	−131.9 (9)	C10—N1—C14—C15	179.2 (11)
N2—Bi1—N1—C10	172.5 (10)	Bi1—N1—C14—C15	0.9 (13)
O4—Bi1—N1—C10	94.5 (10)	C12—C13—C14—N1	−2(2)
O5^i^—Bi1—N1—C10	−115.4 (10)	C12—C13—C14—C15	177.4 (14)
O7—Bi1—N1—C10	23.1 (11)	C19—N2—C15—C16	−0.7 (17)
O10—Bi1—N1—C10	−45.8 (10)	Bi1—N2—C15—C16	156.1 (10)
C9^i^—Bi1—N1—C10	−121.7 (10)	C19—N2—C15—C14	178.1 (10)
O3—Bi1—N2—C15	166.9 (9)	Bi1—N2—C15—C14	−25.1 (13)
O6^i^—Bi1—N2—C15	−119.7 (8)	N1—C14—C15—N2	15.8 (15)
N1—Bi1—N2—C15	18.4 (8)	C13—C14—C15—N2	−164.0 (13)
O4—Bi1—N2—C15	112.6 (8)	N1—C14—C15—C16	−165.3 (11)
O5^i^—Bi1—N2—C15	−66.2 (8)	C13—C14—C15—C16	14.9 (19)
O7—Bi1—N2—C15	136.2 (8)	N2—C15—C16—C17	0.1 (19)
O10—Bi1—N2—C15	−38.8 (10)	C14—C15—C16—C17	−178.7 (12)
C9^i^—Bi1—N2—C15	−93.1 (8)	C15—C16—C17—C18	1(2)
O3—Bi1—N2—C19	−36.2 (8)	C16—C17—C18—C19	−2(2)
O6^i^—Bi1—N2—C19	37.1 (8)	C15—N2—C19—C18	−0.1 (17)
N1—Bi1—N2—C19	175.3 (9)	Bi1—N2—C19—C18	−157.0 (9)
O4—Bi1—N2—C19	−90.5 (8)	C17—C18—C19—N2	1.6 (19)

Symmetry codes: (i) *x*, *y*−1, *z*; (ii) *x*, *y*+1, *z*.

### Hydrogen-bond geometry (Å, °)

**Table:**

*D*—H···*A*	*D*—H	H···*A*	*D*···*A*	*D*—H···*A*
O1—H1···O11^iii^	0.82	1.79	2.60 (2)	167
C10—H10···O8	0.93	2.53	3.16 (2)	125
C12—H12···O10^iv^	0.93	2.58	3.51 (2)	179
C13—H13···O6^v^	0.93	2.57	3.350 (19)	142
C17—H17···O2^v^	0.93	2.48	3.260 (19)	142
C18—H18···O3^vi^	0.93	2.58	3.391 (17)	146
C21—H21B···O9^i^	0.96	2.56	3.49 (3)	161

Symmetry codes: (iii) −*x*+1, −*y*+1, −*z*; (iv) *x*+1, *y*, *z*; (v) *x*+1, *y*−1, *z*; (vi) −*x*+1, −*y*, −*z*; (i) *x*, *y*−1, *z*.
